# Deterioration of Performance Status during Palliative Radiotherapy Suggests a Significant Short Survival Duration: Indicating the Necessities for Considering Radiotherapy Discontinuation

**DOI:** 10.3390/curroncol31040133

**Published:** 2024-03-27

**Authors:** Hitoshi Maemoto, Kazuaki Kushi, Isoko Owan, Takuro Ariga, Joichi Heianna, Akihiro Nishie

**Affiliations:** 1 Division of Radiation Oncology, NHO Okinawa Hospital, Okinawa 901-2214, Japan; 2 Department of Radiology, Graduate School of Medical Science, University of the Ryukyus, Okinawa 903-0213, Japan; arigatak@med.u-ryukyu.ac.jp (T.A.); jana1150@nantoku.org (J.H.); nishie_a@med.u-ryukyu.ac.jp (A.N.); 3 Division of Palliative Care, NHO Okinawa Hospital, Okinawa 901-2214, Japan; 4 Division of Pulmonary Medicine, NHO Okinawa Hospital, Okinawa 901-2214, Japan; owan.isoko.du@mail.hosp.go.jp; 5 Health Information Management Center, University of the Ryukyus Hospital, Okinawa 903-0213, Japan

**Keywords:** radiotherapy, performance status, palliative care, cancer

## Abstract

Discontinuation of palliative radiotherapy due to a patient’s declining general condition poses a clinical dilemma for palliative care physicians. This study aimed to investigate the survival duration of patients whose performance status (PS) deteriorated during palliative radiotherapy and inform decisions regarding early treatment discontinuation. We retrospectively analyzed data from patients referred from our institute’s palliative care department who underwent ≥10 fractions of palliative radiotherapy between March 2017 and December 2021. PS was assessed using the Eastern Cooperative Oncology Group (ECOG) scale. Survival duration was calculated from the final day of palliative radiotherapy to death using the Kaplan–Meier method. A total of 35 patients underwent palliative radiotherapy. Seven (20%) experienced deterioration in ECOG PS during treatment. Their median survival duration was significantly shorter at 22 days (95% confidence interval: 1–94 days) compared to 125 days (95% confidence interval: 82–150 days) for the 28 patients whose PS remained stable (*p* = 0.0007). Deterioration in ECOG PS during palliative radiotherapy signifies a markedly shorter survival duration. Careful assessment of a patient’s condition throughout treatment is crucial, and early discontinuation should be considered if their general health worsens rather than strictly adhering to the initial schedule.

## 1. Introduction

Radiotherapy is a cornerstone of palliative oncology care [1,2,3,4,5,6,7,8,9,10,11,12,13], serving a vital role for a significant portion of cancer patients [9,10,11]. Common palliative radiotherapy regimens include 30 Gy in 10 fractions (30 Gy/10 fr), 24 Gy/6 fr, 20 Gy/5 fr, and 8 Gy/1 fr [14].

Recent studies have demonstrated that short courses like 20 Gy/5 fr and 8 Gy/1 fr for specific palliation can be as effective as 30 Gy/10 fr [3,15,16,17]. This makes them attractive for patients with limited prognoses, preserving their precious time. However, studies also show significantly higher recurrence rates for short regimens compared to 30 Gy/10 fr, 37.5 Gy/15 fr, or 40 Gy/20 fr [3,18,19]. Therefore, for patients with anticipated survival beyond several months, higher-dose regimens like 30 Gy/10 fr or more are preferred.

Determining treatment strategies in palliative radiotherapy hinges on an accurate assessment of survival prognosis. Several prognostic models exist, many incorporating performance status (PS) as a factor [13,20,21,22,23,24,25,26,27,28]. However, predicting survival at the time of choosing a regimen is often challenging, with oncologists tending to overestimate survival duration [29,30]. One report even found that nearly 20% of patients receiving radiotherapy in their final 30 days had spent over 10 days in treatment [31].

In practice, we often encounter patients whose initially predicted survival of several months led to a 30 Gy/10 fr or higher regimen but whose PS then deteriorated during treatment. This raises a dilemma for palliative care physicians: should they continue or discontinue radiotherapy when PS worsens?

Delivering bad news about treatment discontinuation can be emotionally taxing for both patients and physicians [32,33]. This burden is compounded when palliative care physicians hesitate to challenge or discontinue regimens prescribed by radiation oncologists due to insufficient communication because, generally, physicians tend to hesitate to alter decisions made by other specialists [34]. Additionally, concerns about the patient’s condition worsening without treatment or fear of patient and family misunderstanding may cloud the decision-making process [34,35].

Ideally, decisions regarding palliative care, including discontinuation of palliative radiotherapy, should be achieved collaboratively through comprehensive discussions involving patients, family caregivers, palliative care physicians [36,37], and radiation oncologists. However, realizing this ideal within a clinical setting can pose challenges. Substantial disparities in knowledge may exist between patients and medical practitioners. Communication between palliative care physicians and radiation oncologists may be constrained, for example, due to the limited overlap in their work schedules at the facility.

While there are hurdles to discontinuing treatment, as mentioned above, radiotherapy is a local therapy, and continuing it usually does not pose a great physical burden for patients. With these factors in mind, the decision to discontinue may be challenging in certain situations.

Despite its importance, discontinuation of palliative radiotherapy has received limited attention in the literature. This study aims to fill this gap by investigating the survival outcomes of patients with and without PS deterioration during 30 Gy/10 fr or longer regimens. Our findings hope to provide valuable insights for informing decisions surrounding treatment discontinuation.

## 2. Materials and Methods

### 2.1. Ethical Statement

This study was approved by the institutional review board of NHO Okinawa Hospital (Approval Number: 2023-14). This study was conducted in accordance with the principles of the Declaration of Helsinki. The requirement for written informed consent was waived based on the fact that all eligible patients were deceased or unreachable.

### 2.2. Patients

We retrospectively analyzed data from patients referred by the Palliative Care Department of NHO Okinawa Hospital between March 2017 and December 2021. All patients had undergone palliative radiotherapy for malignant tumors at our institute. PS was assessed using the Eastern Cooperative Oncology Group (ECOG) scale.

Patients who received <10 fractions of radiotherapy, were under 20 years old, had a non-tumor cause of death, or had an initial ECOG PS of 4 were excluded. To account for potential day-to-day fluctuations in PS, such as those caused by increased opioid doses, we reviewed medical records from three days before radiotherapy initiation to three days after completion. Palliative care nurses and physicians kept sufficient medical records daily, facilitating retrospective assessment of PS. In addition, radiation oncologists documented PS directly in the medical records at initial consultations and weekly during the course of palliative radiotherapy. None of the patients were receiving or scheduled for systemic therapy (chemotherapy, targeted drugs, or immune checkpoint inhibitors) at the time of radiotherapy referral.

### 2.3. Radiotherapy

Palliative radiotherapy utilized 6 MV or 10 MV photons or a 6 MeV electron beam from a PRIMUS KD2-7467 system (Siemens, Berlin, Germany). The treatment planning was conducted utilizing Xio (Elekta, Stockholm, Sweden). Radiotherapy was performed with a three-dimensional conformal technique in all cases. Intensity-modulated radiation therapy was not used. Treatment began within five days of referral from the palliative care department, with regimens tailored by the attending radiation oncologist based on each patient’s condition and tumor status.

### 2.4. Statistical Analysis

Survival duration was calculated from the final day of palliative radiotherapy to death using the Kaplan–Meier method. We chose this endpoint to focus on the time between the last treatment and the patient’s passing. For patients receiving palliative radiotherapy for multiple lesions with different schedules, only the last course was included. The log-rank test assessed the statistical significance of survival differences between groups, while Welch’s test compared the mean survival days. Statistical significance was set at *p* < 0.05. All analyses using EZR ver. 1.41 (Saitama Medical Centre, Jichi Medical University, Saitama, Japan), a graphical user interface for R (R Foundation for Statistical Computing, Vienna, Austria). This modified version of R commander adds commonly used biostatistics functions [38].

## 3. Results

**Figure 1:**
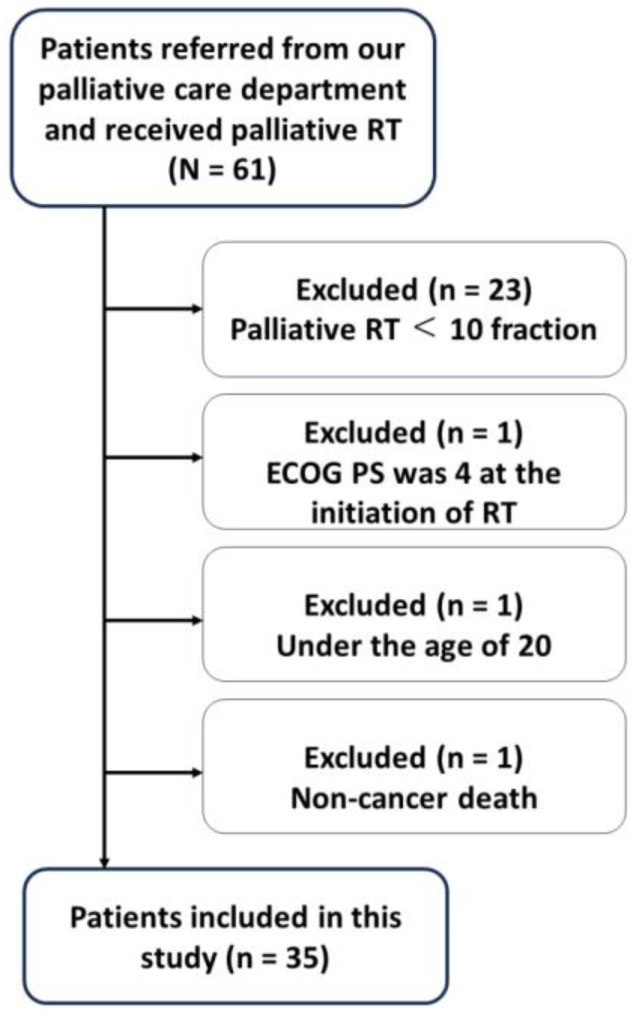
Flow chart of the patients included in this study. RT: radiotherapy, ECOG PS: Eastern Cooperative Oncology Group performance status.

During the study period, 61 patients received palliative radiotherapy referrals. However, only 38 met the inclusion criteria of having a scheduled regimen exceeding 10 fractions. Three patients were excluded due to age (<20), non-malignancy cause of death, or initial ECOG PS of 4. This resulted in a final study cohort of 35 patients (Figure 1).

Table 1 summarizes their characteristics. At the start of radiotherapy, 11 patients had good performance status (ECOG PS of 1); 19 showed moderate limitations (ECOG PS of 2), and five displayed significant limitations (ECOG PS of 3). The primary lesions varied as follows: Head and Neck (*n* = 6); Lung (*n* = 4); Breast (*n* = 3); Prostate (*n* = 1); Esophagus (*n* = 1); Stomach (*n* = 1); Rectum (*n* = 4); Pancreas (*n* = 2); Gallbladder (*n* = 1); Kidney (*n* = 1); Uterine (*n* = 5); Ovary (*n* = 1); Vulva (*n* = 1); and soft tissue sarcoma (*n* = 4). The status of multiple organ metastases was as follows, except for one patient with unknown distant metastasis status: 15 of 34 patients (38.2%) had multiple lung metastases; 4 of 34 patients (11.8%) had multiple liver metastases; and 10 of 34 patients (29.4%) had multiple bone metastases.

The target lesion for palliative radiotherapy was the primary tumor site in 18 cases, accounting for about half of the cases (51.4%), followed by bone metastasis in 12 cases (34.3%), lymph node metastasis in 3 cases (8.6%), and lung metastasis in 2 cases (5.7%).

The most frequently used radiotherapy regimen in this study was 30 Gy/10 fr, administered to 21 patients (60.0%). The selection of radiotherapy regimens was determined by attending radiation oncologists, depending on the evaluation of the tumor and patient status, resulting in the utilization of various dose fractionations in this study.

The treatment regimens, excluding 30 Gy/10 fr and their corresponding target site, were as follows: 21.6 Gy/12 fr (*n* = 2) for pelvic lesions; 25 Gy/10 fr (*n* = 1) for lung metastasis; 30 Gy/15 fr (*n* = 2) for head and neck and breast tumors; 36 Gy/12 fr (*n* = 1) for soft tissue sarcoma; 39 Gy/13 fr (*n* = 3) for soft tissue sarcoma, head and neck tumor, and bone metastasis; 40 Gy/20 fr (*n* = 2) for bone metastases; 42 Gy/14 fr (*n* = 1) for breast tumor; 45 Gy/15 fr (*n* = 1); and 50 Gy/25 fr (*n* = 1) for head and neck tumors.

The reasons for the five cases in which the biologically effective dose was less than 30 Gy/10 fr were as follows: the lesions in each case of pelvic region and lung metastasis exhibited considerable size, prompting concerns regarding potential adverse events. The dose was reduced akin to standard fractionation for one patient each in the pelvic region and head and neck, as further radiation therapy was under consideration, although no additional radiotherapy was actually performed. Additionally, in one case of re-irradiation for head and neck, the dose was reduced. Among the 35 cases in this study, only one was a case of re-irradiation. The last course was included in this study as described within the Materials and Methods section for the case of re-irradiation.

**Figure 2:**
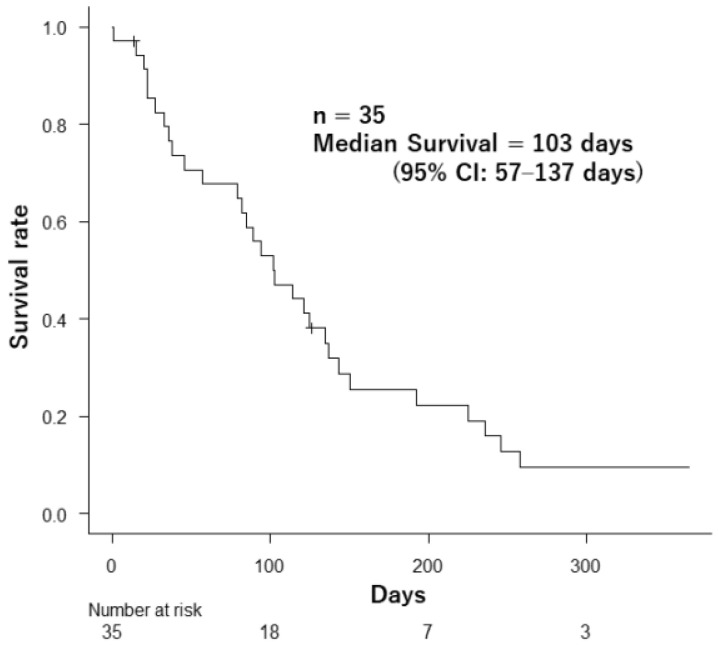
Survival after palliative radiotherapy in 35 patients.

By the final follow-up, 33 patients (94.2%) had passed away. The overall median survival for the group was 103 days (95% confidence interval [CI]: 57–137 days), as illustrated in Figure 2.

**Figure 3:**
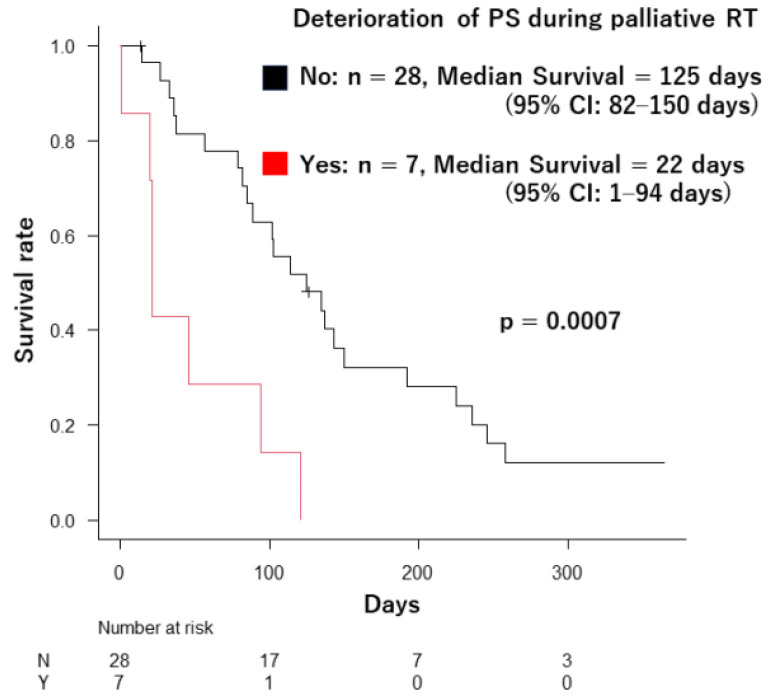
The difference in survival duration between the patients whose ECOG PS deteriorated during palliative radiotherapy and the patients who did not.

A critical finding emerged during this study: seven patients (20%) experienced a decline in their ECOG PS during palliative radiotherapy (PS deterioration from 1 to 2, two patients; from 2 to 3, three patients; from 1 to 3, one patient; and from 2 to 4, one patient). Their median survival was significantly shorter at 22 days (95% CI: 1–94 days) compared to the 125 days (95% CI: 82–150 days) observed in patients with stable PS (*p* = 0.0007) (Figure 3).

A similar analysis revealed consistent patterns when comparing patients based on the severity of decline: four patients whose PS worsened from 1–2 to 3–4 had a median survival of 33 days (95% CI: 1–NA days) compared to 117 days (95% CI: 82–143 days) observed in the remaining 31 patients (*p* = 0.004). A detailed analysis of the degree of PS deterioration was not performed in this study due to the small cohort size.

The ECOG PS at the start of palliative radiotherapy did not significantly influence survival duration. Patients with ECOG PS 1 (*n* = 11) had a median survival of 94 days (95% CI: 22–236 days); those with PS 2 (*n* = 19) lived a median of 114 days (95% CI: 36–137 days), and PS 3 patients (*n* = 5) survived a median of 89 days (95% CI: 15–NA) (*p* = 0.731).

In one case, palliative radiotherapy was stopped early due to disease progression worsening the patient’s condition (planned: 30.6 Gy/17 fractions, delivered: 21.6 Gy/12 fractions). This patient was also included in the analyses. Seven of the 35 patients died within 30 days of completing or stopping treatment. Four of these seven experienced declining ECOG PS during radiotherapy, while the remaining three maintained stable status. The average age of the deceased patients within 30 days (67.3 years, standard deviation [SD] = 12.4) was lower than the surviving group (75.6 years, SD = 12.1), although the difference was not statistically significant (*p* = 0.144).

An analysis of survival duration based on the presence or absence of multiple lung, liver, and bone metastases was conducted, excluding one case with unknown distant metastasis status. The analysis of brain metastases was omitted due to the limited number of patients with documented brain lesions in this cohort.

No significant decrease in survival duration was observed in any of the groups based on the presence or absence of multiple lesions: lung (Yes: *n* = 15, median 82 days [95% CI: 36–103 days] vs. No: *n* = 19, 123 days [95% CI: 27–236 days], *p* = 0.089); liver (Yes: *n* = 4, 42 days [95% CI: 1–NA days] vs. No: *n* = 30, 103 days [95% CI: 79–143 days], *p* = 0.085); and bone (Yes: *n* = 10, 82 days [95% CI: 1–102 days] vs. No: *n* = 24, 123 days [95% CI: 46–150 days], *p* = 0.217).

Survival duration by target site was as follows: primary site (*n* = 18), with a median of 124 days (95% CI: 46–236 days); bone metastasis (*n* = 12), with a median of 85 days (95% CI: 27–121 days); lymph node metastasis (*n* = 3), with a median of 57 days (95% CI: 38–NA); and lung metastasis (*n* = 2), with a median of 184 days (95% CI: 143–NA). No significant difference in survival duration was observed between 18 patients who received palliative RT for the primary site (median, 124 days) and the 17 patients who received RT for the non-primary site (median, 94 days [95% CI: 36–125 days]) (*n* = 0.213). Similarly, no significant difference was observed between the 12 patients who received RT for bone metastasis (median, 85 days) and the 23 patients who received RT for non-bone metastatic lesion (median, 135 days [95% CI: 57–192 days]) (*p* = 0.179).

In the analysis based on the regimen, the survival duration was significantly decreased in five patients who underwent palliative RT with a biologically effective dose of less than 30 Gy/10 fr (median, 21 days [95% CI: 15–NA days]) compared to 30 patients who received RT with 30 Gy/10 fr or more (median, 114 days [95% CI: 82–137 days]) (*p* = 0.036). Although no statistically significant difference was evident, the median survival duration among five patients who received 40 Gy or more was 192 days (95% CI: 82–NA days), whereas in 30 patients who received less than 40 Gy, it was 94 days (95% CI: 38–135 days) (*p* = 0.161).

## 4. Discussion

This study reveals the following finding: in patients undergoing palliative radiotherapy, a deteriorating ECOG PS score during treatment was associated with a significantly shorter survival time, with a median of 22 days compared to stable scores. This highlights the importance of dynamically adjusting the treatment plan based on the patient’s changing condition. Instead of rigidly adhering to the initial schedule, close monitoring and consideration of early discontinuation when the patient’s general health worsens can offer a more patient-centered approach.

As acknowledged in the Introduction, discontinuing palliative radiotherapy can be difficult. Some of the potential hurdles include delivering bad news about treatment changes, potentially conflicting with the radiation oncologist’s initial plan, and the risk of causing patient or family distress.

However, palliative radiotherapy typically takes 3–4 weeks to alleviate symptoms [39]; therefore, timely discontinuation for patients experiencing ECOG PS deterioration during treatment appears appropriate. Their significantly short survival times suggest limited benefit from completing the full course. Moreover, comprehensive palliative care might be a better fit for patients in their final weeks, as the downsides of radiotherapy (adverse effects and disruptions) could outweigh the limited symptom relief [40]. Additionally, re-irradiation remains a viable option for symptom recurrence after discontinuation, even if long-term survival is achieved.

While earlier studies suggest higher mortality within two weeks of palliative radiotherapy completion for younger patients [41], this trend could not be statistically confirmed in our small cohort. However, the observed average age difference between the deceased within 30 days and survival patients (67.3 vs. 75.6 years old, *p* = 0.144) aligns with this trend, hitting a potential preference for more aggressive treatment in younger patients.

Despite the lack of statistical significance, likely due to our sample size, differences in survival based on multiple lung, liver, and bone metastases warrant attention. For patients with multiple metastases not scheduled for systemic therapy, planning a non-short course of palliative radiotherapy (≥10 fractions) may not be optimal, as their prognosis is expected to be relatively short. While radiation oncologists often prioritize symptomatic relief in palliative care, understanding the patient’s overall burden is crucial to making informed treatment decisions.

This study focused on patients who received irradiation of 10 fr or more, and 30 Gy/10 fr was the most frequently used. The prognosis was significantly reduced in cases where the irradiated dose was less than 30 Gy/10 fr in terms of biologically effective dose (the median survival, 21 vs. 114 days, *p* = 0.036). The reason behind the shorter prognosis was understandable, as the cases receiving the lower dose included the cases of exceedingly large tumors, the cases where additional irradiation was considered but not actually performed, and the cases where re-irradiation was performed. In such cases, a brief course of radiotherapy, such as 20 Gy/5 fr or 8 Gy/1 fr, may have been appropriate. However, during the study period, brief-course palliative radiotherapy was not as prevalent at our institution as it is presently.

Conversely, patients who received irradiation of 40 Gy or more tended to demonstrate a longer median survival duration (the median survival, 192 vs. 94 days). Although no statistically significant difference was observed, likely owing to the limited number of analyses, this trend may have arisen from radiation oncologists perceiving patients with relatively favorable prognoses and consequently prescribing higher doses to them.

This study is limited by its retrospective design, a common feature in palliative care research. The relatively small sample size drawn from a single institution further restricts the generalizability of our findings. The heterogeneity of the patient cohort in terms of primary lesions and baseline status at the start of palliative radiotherapy introduces another element of complexity in drawing specific conclusions for different subgroups. The differences in prognosis depending on the cause of PS deterioration could not be analyzed in this study because there were some cases with insufficient records, although most of the causes of PS deterioration were assumed to be the progression of malignant tumors. Finally, a potential selection bias may have skewed this study toward longer treatment regimens. Since almost all participants were hospitalized in our palliative care ward, the advantages of briefer radiotherapy courses were less relevant in this setting. This might inadvertently lead to a higher-than-average proportion of patients experiencing ECOG PS deterioration during palliative radiotherapy. The therapeutic effects of palliative radiotherapy were not evaluated in this study, given the heterogeneity of the population in terms of symptoms, primary site, treatment target site, and treatment regimen. To address these limitations, large-scale prospective studies would be necessary.

## 5. Conclusions

This study’s findings suggest that ECOG PS deterioration during palliative radiotherapy is significantly associated with short survival duration. This underscores the importance of meticulously evaluating patients’ condition throughout treatment and considering early discontinuation if their general health worsens rather than rigidly adhering to the initial treatment schedule.

## Figures and Tables

**Table 1:** Patient and treatment characteristics (*n* = 35).

Factor	
Age, median (range), years	74 (48–95)
Sex, Female/Male	19/16
ECOG PS, 1/2/3	11/19/5
Primary site	
Head and Neck	6
Lung	4
Breast/Prostate	3/1
Esophagus/Stomach/Rectum	1/1/4
Pancreas/Gallbladder/Kidney	2/1/1
Uterine/Ovary/Vulva	5/1/1
Soft tissue sarcoma	4
Multiple organ metastasis *	
Multiple lung metastases	Yes: 15/No: 19
Multiple liver metastases	Yes: 4/No: 30
Multiple bone metastases	Yes: 10/No: 24
Target lesion for Palliative RT	
Primary site	18
Bone metastasis	12
Lymph node metastasis	3
Lung metastasis	2
RT regimen (classified by BED)	
<30 Gy/10 fr	5
30 Gy/10 fr	21
>30 Gy/10 fr	9

* Evaluation of multiple organ metastases was performed in a total of 34 patients, excluding one patient whose status of distant metastasis was unknown. ECOG PS: Eastern Cooperative Oncology Group Performance Status; RT: radiotherapy; fr: fraction; BED: biologically effective dose.

## Data Availability

The data presented in this study are available on request from the corresponding author. The data are not publicly available due to privacy restrictions.
